# Preparation of molecular imprinted fluorescence sensor based on Er^3+^/ZnS QDs and its selective detection of ciprofloxacin in various matrices

**DOI:** 10.1371/journal.pone.0312156

**Published:** 2024-12-19

**Authors:** Shuang Li, Haijun Wang, Jinghan Wang, Feng Xu, Hongmei Li

**Affiliations:** Qiqihar Medical University, Qiqihar, China; UniCamillus: Saint Camillus International University of Health and Medical Sciences, ITALY

## Abstract

**Background:**

Ciprofloxacin is a widely used antibiotic in medicine and agriculture. It can cause pollution to the environment and food, thereby affecting human health.

**Objective:**

This study proposes the preparation of molecular imprinted fluorescent sensors and their selective detection of ciprofloxacin, with the aim of achieving specific recognition and accurate detection of ciprofloxacin.

**Methods:**

Rare earth metal Er^3+^ is added to ZnS quantum dots to prepare a molecularly imprinted fluorescence sensor (MIP/Er^3+^/ZnS QDs). The effects of substance concentration, pH value, and time on the fluorescence detection intensity are analyzed to determine the optimal fluorescence detection conditions.

**Results:**

Experimental results showed that the sensor accurately detected ciprofloxacin with a detection limit of 31 nmol/L and the linear range of 0.1–10 μmol/L. The sensor had a recovery rate of 99.7% to 103.5% for ciprofloxacin in milk samples, with a relative standard deviation of less than 5%, indicating accurate determination of ciprofloxacin content.

**Conclusion:**

Molecularly imprinted fluorescent sensors have enormous application potential in the monitoring and control of ciprofloxacin, and are of great significance for ensuring environmental and food safety.

## Introduction

Recently, as the increasing severity of environmental pollution and food safety issues, the demand for fast, accurate, and highly selective detection methods has been increasing [1]. Ciprofloxacin, as a broad-spectrum antibiotic widely used in veterinary medicine and pesticides, has attracted widespread attention due to its residual issues [2]. The traditional detection methods for ciprofloxacin mainly contain high-performance liquid chromatography, gas chromatography, and mass spectrometry. However, these methods typically necessitate the use of costly instrumentation and intricate sample preparation procedures, which are also time-consuming [3]. Molecular imprinting technology is a novel detection method based on molecular recognition principles, which forms polymer materials with specific recognition cavities through the interaction between template molecules and functional monomers [4]. This material can selectively adsorb target molecules and achieve detection of target molecules by changing their optical properties. Molecular imprinting technology has been widely used in environmental monitoring and food safety due to its advantages [5]. This study focuses on the selective detection of ciprofloxacin and innovatively combines molecular imprinting technology with fluorescence sensors to prepare a fluorescence sensor with high selectivity and accuracy. This sensor uses ZnS quantum dots (QD) as carriers and introduces rare earth metal Er^3+^ and specific functional monomers to form a polymer material with specific recognition cavities, achieving efficient detection of ciprofloxacin. In comparison to conventional detection techniques, this technology offers the benefits of reduced cost, enhanced stability, and repeated use, thereby providing a rapid, precise, and highly selective detection method for environmental monitoring and food safety applications. This research result not only has important application value for the monitoring and control of ciprofloxacin but also provides new ideas and methods for the detection of other antibiotics, with broad application prospects.

Molecular imprinting fluorescence sensor is a sensor that combines molecular imprinting technology and fluorescence sensing technology to detect and analyze target molecules through specific recognition and fluorescence signal changes. It has high sensitivity and selectivity, and is suitable for environmental monitoring, food safety, and other fields. Chen et al. proposed an environmentally friendly molecularly imprinted fluorescence sensor based on novel near-infrared fluorescent carbon dots and metal organic frameworks for rapid determination of trypsin. The sensor exhibited a linear relationship within the range of 0.05 to 9.0 ng/L, including a detection limit (DL) of 8.8 pg/L. Within 1.5 minutes, trypsin could be selectively recognized with recoveries of 95.3%-104.1% and 97.7%-105.2%, respectively [6]. Luo et al. has designed a ratio fluorescence sensor (MIR sensor) based on calcium fluoride QD for sensitive detection of 5-hydroxymethylfurfural (HMF). This sensor was constructed using calcium fluoride QD and CdTe QD with purple, blue, green, and yellow emissions, achieving high selectivity through specific molecularly imprinted polymers (MIPs) [7]. Sunayama et al. proposed a fluorescence sensing system based on MIPs that can simultaneously detect tumor marker proteins. Fluorescent labeled prostate specific antigen (PSA) and alpha fetoprotein (AFP) imprinted polymer nanocavities were prepared using dual imprinting and multi-step post imprinting modification methods. This sensing system could convert protein binding events into specific fluorescence signals with low DL [8]. Singh et al. developed a novel organic silane with 2-aminofluorenyl triazole linkage and found that probe 6a has high selectivity for Fe (III) ions. In-vitro and molecular docking studies have shown that probe 6a may serve as an inhibitor of leishmaniasis proteins, providing useful strategies for the treatment of this disease [9]. Leng et al. prepared a novel molecularly imprinted photopolymer through CsPbBr_3_ QD and TpPa-2 substrate. The data showed that the polymer exhibited good linearity towards patulin (PAT) in the range of 0.2–20 ng/mL, with a DL of 0.027 ng/mL [10].

Ciprofloxacin is a broad-spectrum antibiotic commonly utilized for treating infections from bacteria. Perumal et al. synthesized a diarylimidazole derivative 2- (2,4-difluorophenyl) -3- (3,5-dimethylphenyl) -4, 5-diphenyl-1H-imidazole (DFMPI) composed of electron withdrawing and electron donating substituents, and characterized it. This compound exhibited good characteristics in iron ion selective sensing. Theoretical calculations indicated that DFMPI exhibited nonlinear optical behavior and charge transfer within the molecule, which has potential applications in the fluorescence detection of ciprofloxacin [11]. Dang et al. successfully developed a fluorescence off-off-off nanosensor by using N-doped carbon dots as sensing probes. This sensor could detect Cu^2+^ ions and ciprofloxacin antibiotics with ultra sensitivity. The linear response range of Cu2^+^ ions was 0.01–0.35μM, with a DL of 0.076μM, while the linear response range of ciprofloxacin was 0.05–1 and 1–50μM, with a DL of 0.4nM [12]. Stojanovic et al. found through fluorescence and absorption spectroscopy that fluoroquinolone drugs sparfloxacin, levofloxacin, and ciprofloxacin have an impact on the interaction in tigecycline and human serum albumin (HSA). Sparfloxacin and ciprofloxacin grew the binding constant of the HSA tigacyclin system, while levofloxacin slightly decreased the binding constant [13].

In summary, molecular imprinting fluorescence sensors, combined with molecular imprinting technology and fluorescence sensing technology, demonstrate the high sensitivity and selectivity of detecting and analyzing ciprofloxacin in fields such as environmental monitoring and food safety. However, existing molecularly imprinted fluorescence sensors still face some challenges in practical applications. Firstly, the preparation process of sensors is relatively complex, involving numerous materials and experimental techniques. How to simplify the preparation process and improve the repeatability and stability of sensors is an important research topic at present. Secondly, although molecularly imprinted fluorescence sensors have been successfully developed for various biomolecules, the detection of molecules with complex structures and biological environments remains a significant challenge. Improving the detection sensitivity and selectivity of such molecules is another challenge in the research of molecularly imprinted fluorescence sensors. To further enhance the effectiveness of ciprofloxacin detection, this study proposes the preparation of molecular imprinted fluorescent sensors and their selective detection of ciprofloxacin, with the aim of achieving specific recognition and accurate detection of ciprofloxacin. This also provides a reliable analytical tool for areas such as environmental monitoring and food safety.

## Materials and methods

### Experimental instruments and reagents

The instruments required for the experiment include transmission electron microscopy (TEM), scanning electron microscopy (SEM), energy spectrometer, ultraviolet (UV) spectrophotometer, fluorescence spectrophotometer, Fourier infrared spectrometer, collector type constant temperature heating magnetic stirrer, and ultrasonic cleaner. The combination of these instruments can meet the requirements for the microstructure, surface morphology, elemental composition, spectral characteristics, and sample processing of materials. The necessary instrument information is shown in Table 1.

**Table 1:** Instrument information required for the experiment.

Number	Instrument name	Instrument usage	Manufacturer	Instrument model
1	High-resolution TEM	Generate TEM image	JEOL	JEM-2100HR
2	SEM	Generate SEM image	Zeiss, Germany	EVO18
3	UV spectrophotometer	UV spectrum	Shimadzu, Japan	UV-2550
4	Fluorescence spectrophotometer	Fluorescence spectrum	VARLAN, USA	Cary Eclipse
5	Fourier infrared spectrometer	Infrared spectrum	Negoli, USA	Nicolet Nexus 470
6	Collecting type constant temperature heating magnetic stirrer	Heating and stirring	Gongyi Yuhua Instrument Co., Ltd	DF-101s
7	Ultrasonic cleaner	Clean experimental samples	Kunshan Ultrasonic Instrument Co., Ltd	KQ-50E

The reagents required in the experiment include ciprofloxacin hydrochloride monohydrate, sodium sulfide nine hydrate, 3-mercaptopropionic acid, 3-aminopropyltriethoxysilane, tetraethyl silicate, erbium nitrate pentahydrate, zinc sulfate heptahydrate, and other analytical pure chemical reagents. In addition, deionized water is also required for solution preparation and cleaning operations. The necessary main reagents are demonstrated in Table 2.

**Table 2:** Main reagents required for the experiment.

Number	Reagents name	Specifications	Manufacturer
1	Ciprofloxacin (CFX)	Analytical pure	Shanghai Aladdin Reagent Co., Ltd
2	Sodium sulfide decahydrate (Na_2_S•9H_2_O)	Ditto	Ditto
3	3-mercaptopropionic acid (MPA)	Ditto	Ditto
4	3-aminopropyltriethoxysilane (APTES)	Ditto	Ditto
5	Tetraethyl silicate (TEOS)	Ditto	Ditto
6	Erbium nitrate pentahydrate (Er(NO_3_)_3_•5H_2_O)	Ditto	Shanghai McLean Biochemical Technology Co., Ltd
7	Zinc sulfate heptahydrate (ZnSO_4_•7H_2_O)	Ditto	Guangzhou Chemical Reagent Factory
8	Glacial acetic acid	Ditto	Sinopharm
9	Deionized water	Ditto	3M company

### Procedure

#### The synthesis of Er^3+^/ZnS QDs

The steps for synthesizing Er^3+^/ZnS QDs are as follows:

1. Preparation of experimental equipment and reagents: Prepare a 250mL three necked flask, 3-mercaptopropionic acid, zinc sulfate solution, erbium nitrate solution, sodium hydroxide solution, Na_2_S solution, anhydrous ethanol, and other reagents.
2. Add 3-mercaptopropionic acid: First, add 50mL of 0.04mol/L 3-mercaptopropionic acid to a three necked flask.
3. Add zinc sulfate solution: Next, add 5mL of 0.1mol/L zinc sulfate solution to the flask.
4. Add erbium nitrate solution: Then, add 1mL of different concentrations of erbium nitrate solution to the flask.
5. Stir evenly: Stir the solution in the flask evenly.
6. Adjust pH value: Use 1mol/L sodium hydroxide solution for adjusting the pH value of the mixed solution to 11.
7. Nitrogen gas injection: Apply nitrogen gas to the flask for 30 minutes to fill it with nitrogen gas.
8. Add Na_2_S solution: Quickly add 5mL of 0.1mol/L Na_2_S solution and vigorously stir for 20 minutes.
9. Aging: Place the solution in air and age at 50°C for 2 hours.
10. Cooling: Allow the solution to cool after being left to cool.
11. Precipitation: Add 70 mL of anhydrous ethanol to precipitate the QD.
12. Centrifuge: Use a centrifuge to centrifuge the solution for 10 minutes (2000 r/min).
13. Washing: After removing the upper clear liquid, wash the solid three times with anhydrous ethanol.
14. Drying: Finally, place the sample in a 60°C oven for drying.

#### The synthesis of MIP/Er^3+^/ ZnS QDs

**Fig 1:**
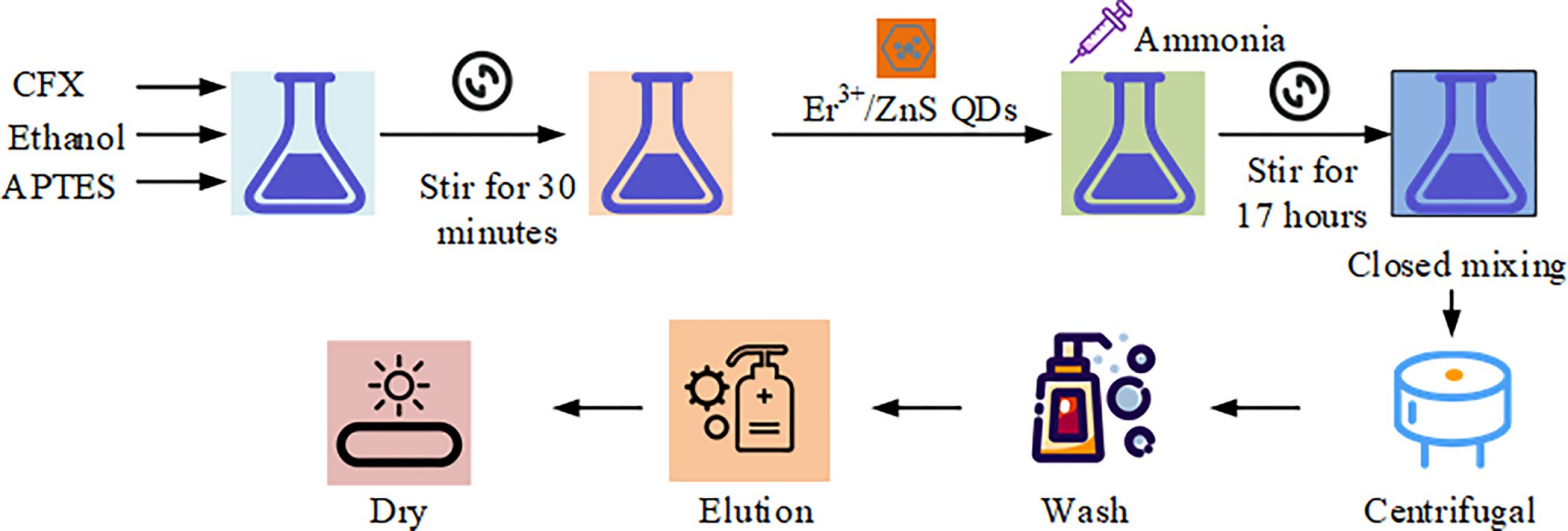
The process of synthesizing MIP/Er^3+^/ZnS QDs.

The steps for synthesizing MIP/Er^3+^/ZnS QDs are as follows:

1. Preparation materials: Prepare 50mg of CFX solid, 10 mL of anhydrous ethanol, and 0.5 mL of APTES.
2. Preparation of mixed solution: Add ciprofloxacin solid, anhydrous ethanol, and APTES to a single neck flask and stir for 30 minutes.
3. Add TEOS: Add 1mL of TEOS to the above mixed solution and continuously stir for 5 min.
4. Add Er^3+^/ZnS QDs solid: Add 50 mg of Er^3+^/ZnS QDs solid to the above mixed solution.
5. Add ammonia water: Add 0.5 mL of 5% ammonia water to the above mixed solution.
6. Closed stirring: Mix the mixed solution tightly for 17 hours.
7. Centrifuge: Centrifuge the obtained solution (2000 r/min).
8. Washing: Wash the solid with anhydrous ethanol three times.
9. Drying: Dry the washed solid in a 60°C drying oven.
10. Extraction solution treatment: Use a mixed solution of ethanol/0.1mol/L acetic acid (4:1) as the extraction solution, and sonicate for 15 minutes.
11. Elution: Elute the MIP/Er^3+^/ZnS QDs until no template molecule ciprofloxacin is detected in the eluent.
12. Drying: Dry the eluted solution to obtain MIP/Er^3+^/ZnS QDs.
13. Repeat the above process to obtain Er^3+^-doped ZnS QD (NIP/Er^3+^/ZnS QDs) coated with template free MIPs, without the addition of ciprofloxacin. The process of synthesizing MIP/Er^3+^/ZnS QDs is shown in Fig 1.

### Preparation of standard solution

The steps for preparing a standard solution are as follows:

1. Prepare a disodium hydrogen phosphate (DHP) citrate buffer solution: Weigh an appropriate amount of DHP and citric acid in a certain proportion, add an appropriate amount of deionized water, stir to dissolve, and adjust the pH value to 5.
2. Weigh 36.8 mg of ciprofloxacin hydrochloride monohydrate and add it to a pH = 5 DHP citrate buffer solution, stir and dissolve it.
3. Transfer the dissolved solution to a 100 mL volumetric flask.
4. Add DHP citrate buffer solution to the mark to obtain a standard stock solution of difloxacin with a concentration of 1.0*10^-3^mol/L.
5. Accurately take an appropriate amount of difloxacin standard stock solution and a certain amount of molecularly imprinted QD, and add them to a DHP citrate buffer solution.
6. Dilute with DHP citrate buffer solution to obtain a series of different concentrations of sulfamethoxazole standard solutions, each containing the same QD.

#### Fluorescence detection

The process of preparing fluorescence detection solution is as follows:

1. Prepare 12 concentrations of CFX standard ethanol solutions with concentrations of 0, 6, 15, 30, 50, 80, 120, 250, 500, 1000, and 1500 nM.
2. Weigh 50 mg of MIP/Er^3+^/ZnS QDs, add them to 100 mL of ethanol, and disperse them using ultrasonic vibration.
3. Take 12 prepared ciprofloxacin solutions and pour them into 10 mL colorimetric tubes.
4. Add an equal volume of MIP/Er^3+^/ZnS QDs dispersion to each colorimetric tube, disperse using ultrasound, and let stand for a few minutes.
5. Transfer the test solution to a colorimetric dish for fluorescence measurement. The relationship between the quenching efficiency of MIP/Er^3+^/ZnS QDs and the concentration of CFX in fluorescence detection is shown in Eq (1).

$$K_{sv}C=1-F_{0}/F$$ (1)

In Eq (1), the initial fluorescence intensity (FI) without adding CFX is *F*_0_, the FI after adding CFX is *F*, the quenching constant is *K*_*sv*_, and the concentration of CFX is *C* [14].

#### Preparation of test samples

The process of preparing test samples is as follows:

1. Prepare milk/water samples and acetonitrile, and take 5mL of milk/water samples and 5mL of acetonitrile, respectively.
2. Add the milk/water sample and acetonitrile to a 15 mL centrifuge tube.
3. Use an oscillator to oscillate the mixture in the centrifuge tube for 5 minutes to fully mix.
4. Centrifuge the centrifuge tube at a speed of 2000 rpm for 15 minutes to separate the supernatant and precipitate.
5. Transfer the supernatant to another container.
6. Evaporate the supernatant to dryness at 50°C to remove solvent.
7. Dissolve the dried extract again in 10mL buffer solution to obtain a solution sample.
8. Mix the solution sample with MIP/Er^3+^/ZnS QDs solution.
9. Perform fluorescence analysis on the mixed solution using a fluorescence analyzer to detect the presence of the target substance [15].

#### Statistical analysis and optimization

*Statistical analysis*. The experiment was repeated multiple times and the average value was calculated to reduce experimental errors. At the same time, multiple parallel measurements were conducted on the standard solution and actual samples to maintain consistency in the results.

*Optimization of experimental conditions*. The experiment optimizes the pH value, ion concentration, and other conditions to achieve optimal sensitivity and specificity of the sensor. At the same time, the optimal extraction solvent and sample processing method in the experiment are used to achieve the maximum recovery rate of the target analyte.

## Results and discussion

### Morphological characterization of materials

**Fig 2:**
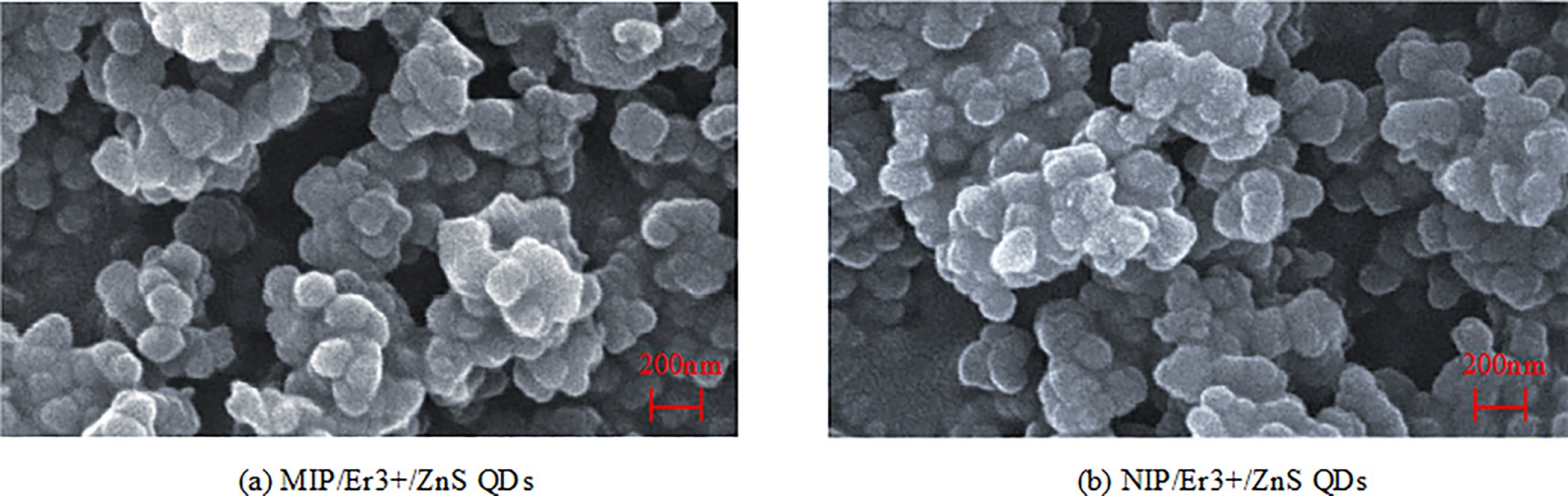
SEM images of MIP/Er^3+^/ZnS QDs and NIP/Er^3+^/ZnS QDs. (a): MIP/Er^3+^/ZnS QDs; (b): NIP/Er^3+^/ZnS QDs.

Morphological characterization is crucial for the application of nanoparticles, as their morphology and distribution can affect their optical, electrical, and magnetic properties [16]. Therefore, morphological characterization can help to understand the structural characteristics of materials and provide a basis for further research and application [17]. The morphological characterization of the material can be displayed through SEM. The SEM images of MIP/Er^3+^/ZnS QDs and NIP/Er^3+^/ZnS QDs are shown in Fig 2.

Fig 2 shows that both MIP/Er^3+^/ZnS QDs and NIP/Er^3+^/ZnS QDs materials exhibit spherical morphology and are uniformly distributed in the sample. This means that the nanoparticles of these two materials exhibit a spherical appearance and are evenly distributed in the sample. This spherical shape and uniform distribution are crucial for the application of nanoparticles. The spherical shape can provide a larger specific surface area, thereby increasing the contact area with the surrounding environment and facilitating reaction processes such as adsorption, catalysis, and transport. Secondly, the use of a uniform distribution ensures the dispersion and stability of nanoparticles in the sample, thereby preventing particle aggregation and deposition. This process maintains the performance and activity of the nanoparticles [18]. Therefore, the spherical morphology and uniform distribution of these two materials provide a good foundation for their subsequent experimental analysis.

### Spectral characterization of materials

**Fig 3:**
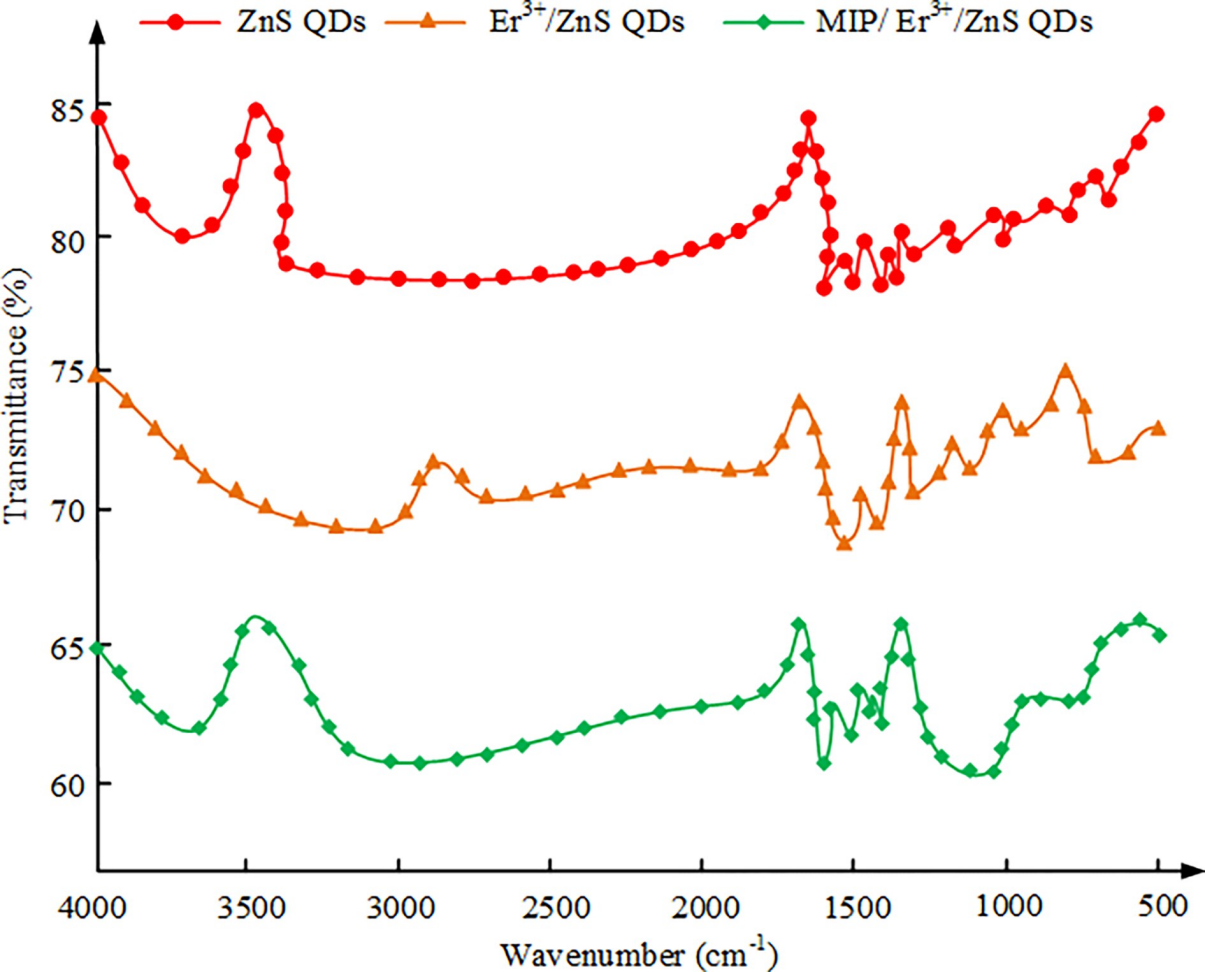
Fourier transform IS of different materials.

Fourier transform infrared spectroscopy is a method used to analyze the infrared spectrum of a substance. This is achieved by exposing the sample to infrared radiation and measuring the intensity of the infrared light absorbed or transmitted by the sample [19]. A spectrogram usually takes the wave number as the horizontal axis as well as the absorbed or transmitted light intensity as the vertical axis. In the spectrogram, the absorption peak represents the specific wavelength at which the sample absorbs infrared light, while the transmission valley represents the transmittance of the sample to infrared light [20]. Fourier transform infrared spectroscopy can be utilized for determining the structure as well as chemical composition of substances, and different chemical bonds and functional groups will generate specific absorption peaks in the infrared spectrum. Therefore, the composition of the sample can be determined by comparing its spectrum with the spectral library of known substances [21]. The Fourier transform infrared spectra (IS) of different materials are shown in Fig 3.

**Fig 4:**
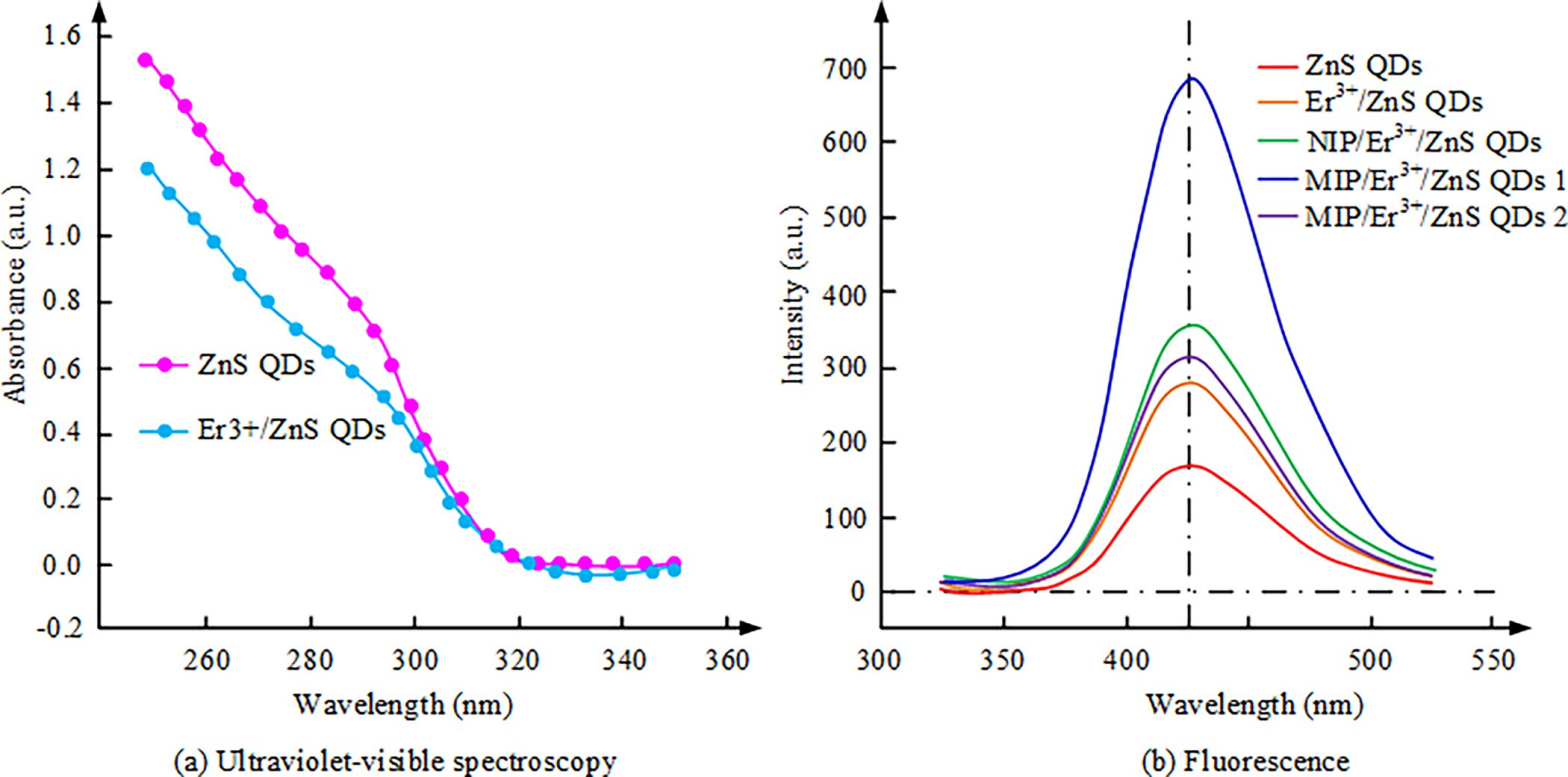
Ultraviolet spectra and FES of different materials. (a): Ultraviolet-visible spectra; (b): Fluorescence.

In Fig 3, by analyzing the Fourier transform IS of ZnS QDs, Er^3+^/ZnS QDs, and MIP/Er^3+^/ZnS QDs, the following conclusions can be drawn. Firstly, the IS of ZnS QDs show three peak positions, namely 1600 cm^-1^, 1400 cm^-1^, and 1050 cm^-1^. These peak positions are caused by the stretching vibration of the carbonyl group on the carboxyl functional group of the protective agent MPA used in the synthesis of QD. Meanwhile, because of the formation of S-Zn covalent bonds, the S-H characteristic absorption peak of MPA thiol functional groups disappears in the range of 2500–2600 cm^-1^. Secondly, the IS of Er^3+^/ZnS QDs shows that the asymmetric stretching vibration and symmetric stretching vibration peaks of carboxyl groups shift to 1680 cm^-1^, 1400 cm^-1^, and 990cm^-1^, respectively. Meanwhile, two absorption peaks appear at 2800 cm^-1^ and 1680 cm^-1^, indicating that Er^3+^ doping enters the ZnS QD. Finally, the IS of MIP/Er^3+^/ZnS QDs shows a characteristic absorption peak at 1070 cm^-1^, which originates from the asymmetric stretching of Si-O-Si in the synthesized MIP [22]. Meanwhile, the vibration of the Si-O bond causes absorption peaks at 464 cm^-1^ and 800 cm^-1^. The UV spectra and fluorescence emission spectra (FES) of different materials are shown in Fig 4.

Fig 4(A) shows the UV-visible spectra of ZnS QDs before and after the addition of Er^3+^. The results in the Fig 4(A) indicate that the maximum UV-visible absorption wavelength of ZnS QDs without the addition of Er^3+^ is approximately 285 nm. However, when Er^3+^ is doped into ZnS QDs, the maximum absorption wavelength undergoes a red shift. Based on these results, the study infers the effect of Er^3+^ addition on the material. The doping of Er^3+^ may lead to changes in the band structure, thereby affecting the optical properties of the material. Specifically, the doping of Er^3+^ may cause a rearrangement of energy bands, leading to changes in band width and thus affecting the absorption spectrum of the material. In addition, the doping of Er^3+^ may also cause changes in the energy level structure of the material [23]. Er^3+^ ions have a special energy level structure, and the transitions in their energy levels can lead to specific wavelengths of light absorption. Therefore, when Er^3+^ is doped into ZnS QDs, the change in its energy level structure may lead to a red shift in the absorption wavelength. Fig 4(B) shows the FES of different materials, with labels 1 and 2 after MIP/Er^3+^/ZnS QDs indicating pre-elution and post-elution, respectively. After adding the Er^3+^ to pure ZnS QD, the FI of ZnS QD is enhanced. The FES of NIP/Er^3+^/ZnS QD with synthesized SiO_2_ molecular imprints shows a higher FI. It may be due to the ability of SiO_2_ molecular imprinting to reduce the non-radiative attenuation of the system. The FES of MIP/Er^3+^/ZnS QD before eluting ciprofloxacin is further enhanced compared to NIP/Er^3+^/ZnS QD. The FES of MIP/Er^3+^/ZnS QD after removing ciprofloxacin in the system shows a slightly lower FI than that of NIP/Er^3+^/ZnS QD. This may be caused by a small amount of QD loss during the elution process. In summary, the introduction of Er^3+^ and the synthesis of SiO_2_ molecular imprinting can enhance the FI of ZnS QD. Before eluting ciprofloxacin, the FI of MIP/Er^3+^/ZnS QD is the strongest, but after eluting ciprofloxacin, its FI is slightly lower than that of NIP/Er^3+^/ZnS QD.

### Optimization of fluorescence detection conditions

**Fig 5:**
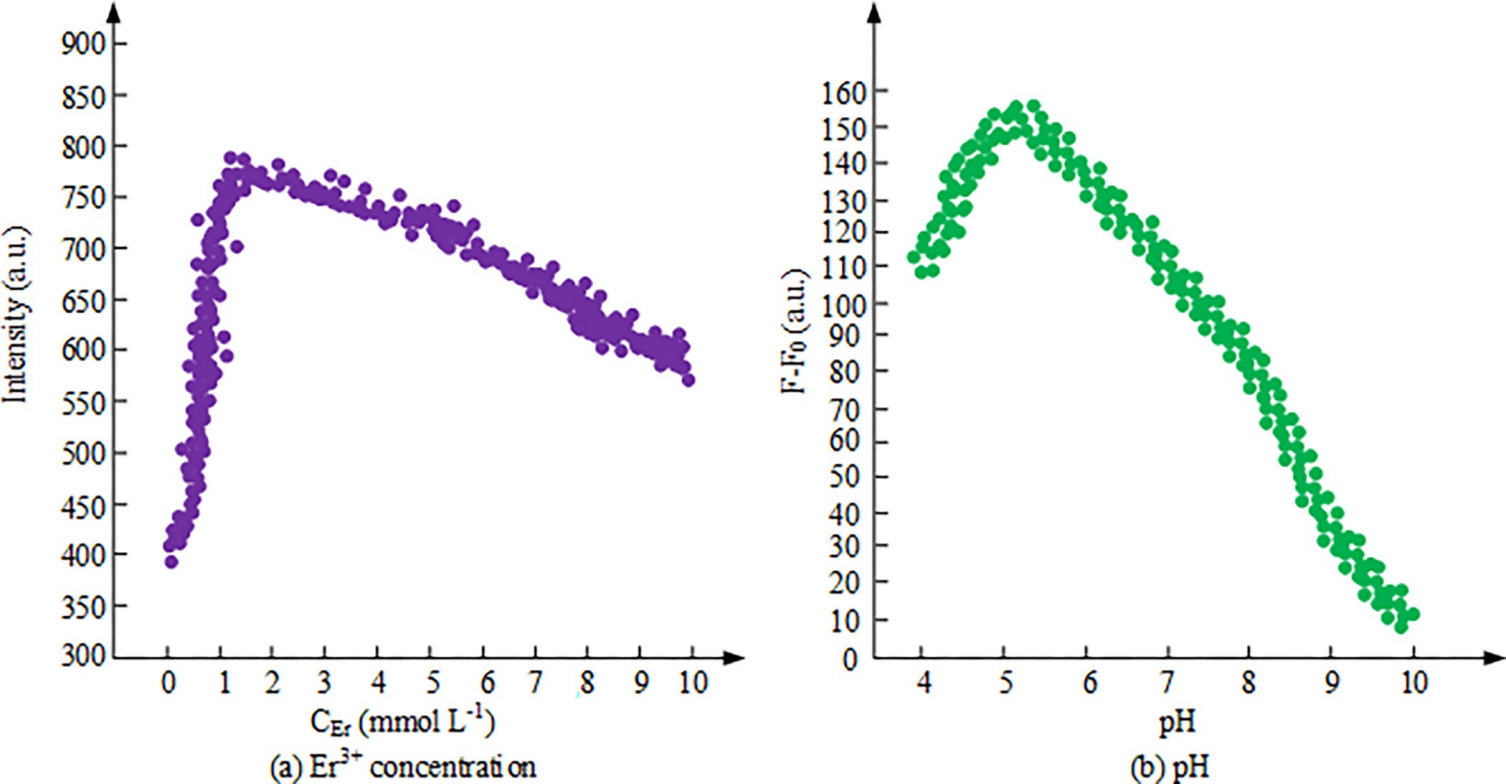
Effects of Er^3+^ concentration and pH value on the FI of MIP/Er^3+^/ZnS QDs.

To optimize the conditions for fluorescence detection, this study analyzes the effects of substance concentration, pH value, and time on fluorescence detection intensity, to determine the optimal fluorescence detection conditions. The effects of Er^3+^ concentration and pH value on the FI of MIP/Er^3+^/ZnS QDs are shown in Fig 5.

**Fig 6:**
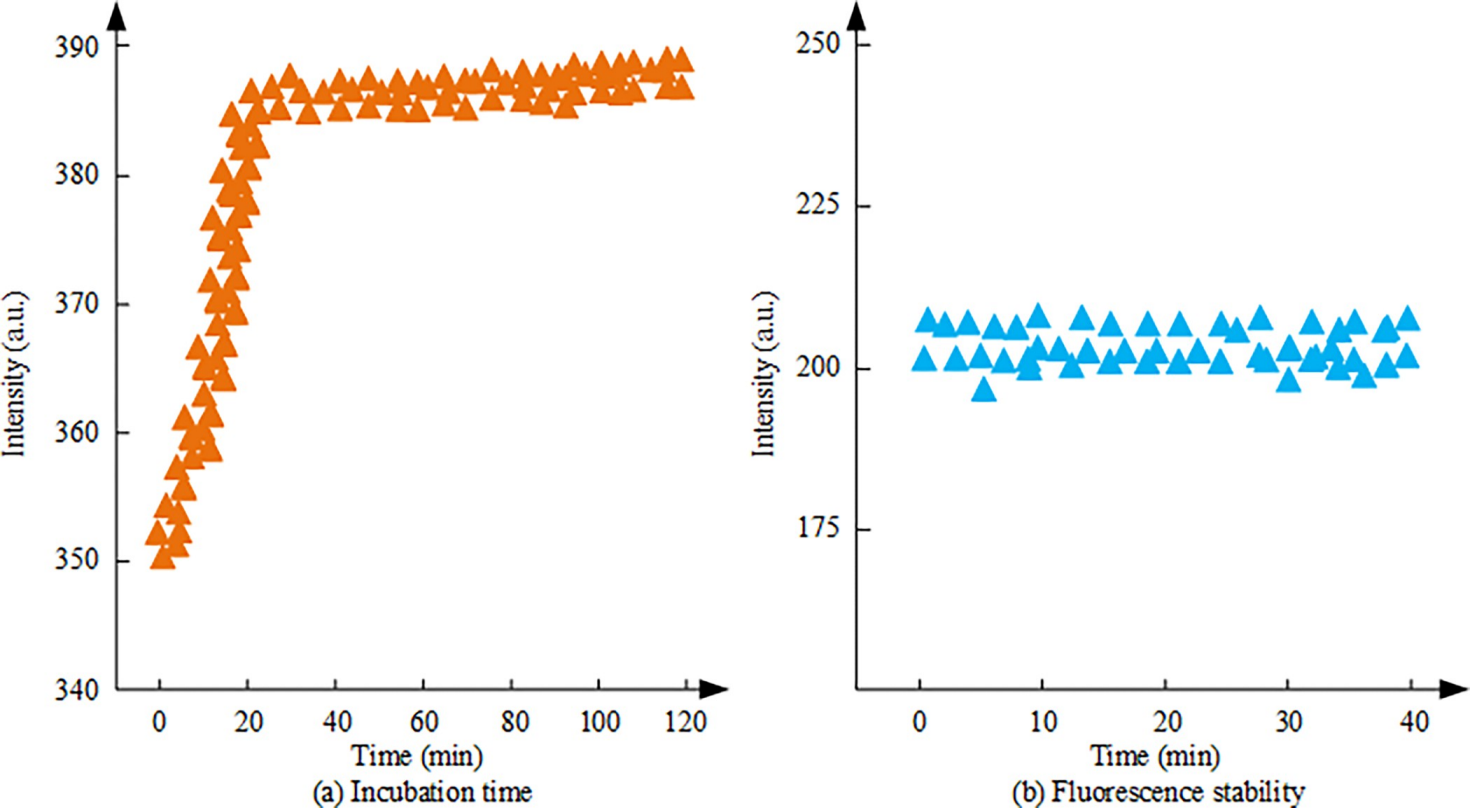
Incubation time and fluorescence stability analysis results. (a): Incubation time; (b): Fluorescence stability.

Fig 5(A) shows the effect of the addition amount of Er^3+^ ions on the FI of ZnS QD. The experimental results indicate that the addition of Er^3+^ ions possesses an essential influence on the FI of ZnS QD. When the concentration of Er^3+^ is low (0.1 mmol/L), the FI is weak. As the concentration of Er^3+^ increases, the FI gradually grows and achieves its maximum value at around 1.2 mmol/L. This is because the energy level structure and energy transfer mechanism of Er^3+^ ions can enhance the fluorescence efficiency of ZnS QD. However, as the concentration of Er^3+^ continues to increase to a higher level (exceeding 1.2mmol/L), the FI begins to gradually decrease. This may be due to the increased interaction between ions caused by the high concentration of Er^3+^, which inhibits the improvement of fluorescence efficiency. Therefore, selecting 1.2mmol/L as the optimal Er^3+^ concentration can achieve the maximum FI. Fig 5(B) shows the effect of different pH values on the FI of MIP/ Er^3+^/ZnS QD. The experimental results indicate that different pH values have a direct impact on the FI of MIP/Er^3+^/ZnS QD. The fluorescence enhancement effect of MIP/Er^3+^/ZnS QD in buffer solutions with different pH values is experimentally measured. It is found that as the pH value gradually increases from a lower range, the FI of the system also increases. The FI reaches its maximum value at around pH5, which is because the acidity and alkalinity of the system are moderate under this pH condition, which is conducive to the interaction between Er^3+^ ions and other components, thereby improving fluorescence efficiency. However, as the pH value continues to increase, the fluorescence enhancement effect of the system gradually weakens, possibly because the excessively high pH weakens the interaction between Er^3+^ ions and other components [24]. In addition, the experiment also investigates the effect of different buffer solution types on the fluorescence of the system, and finds that the FI of the system is the best in the DHP citrate buffer solution. After comprehensive consideration, selecting pH = 5 and a DHP citrate buffer solution as the optimal conditions can obtain the maximum FI of MIP/Er^3+^/ZnS QD. The incubation time and fluorescence stability analysis results are shown in Fig 6.

**Fig 7:**
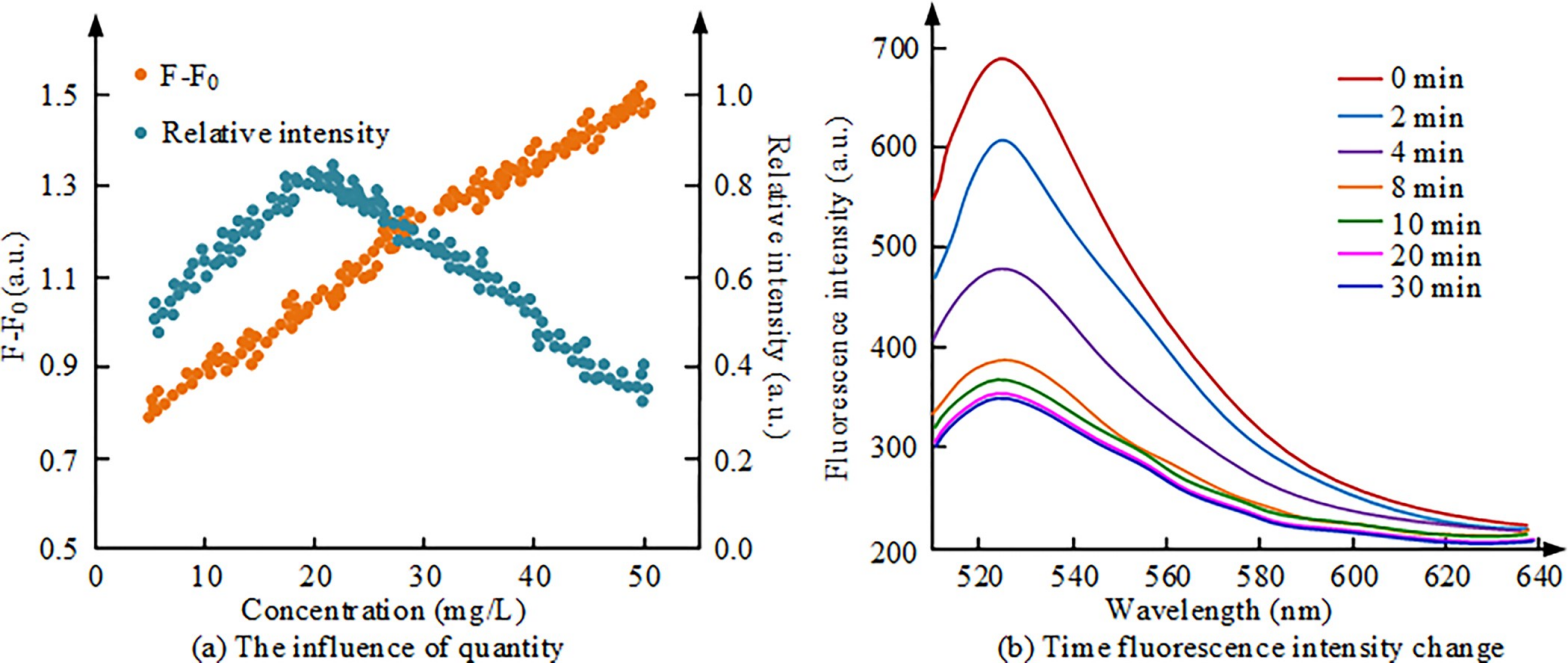
The effect of fluorescence concentration and target binding time on the fluorescence system. (a): The influence of quantity; (b): Time FI change.

Fig 6(A) shows the effect of incubation time on the FI of MIP/Er^3+^/ZnS QDs. The experimental results indicate that different incubation times have an impact on the FI of MIP/Er^3+^/ZnS QDs. The FI of the system increases with time during the incubation time of 0–20 min and reaches its maximum value at 20 min. Subsequent increases in incubation time do not significantly increase the FI. This suggests that within 20 minutes, the analyte can fully bind to the recognition site of the molecularly imprinted membrane to form a stable complex. Further prolonging the incubation time will not result in additional fluorescence enhancement. Therefore, selecting 20 minutes as the optimal incubation time can obtain the maximum FI of MIP/Er^3+^/ZnS QDs. Fig 6(B) shows the detection results of fluorescence stability of MIP/Er^3+^/ZnS QDs. The results demonstrate that the FI of the sensor remains almost stable within 40 minutes. This indicates that within the testing time range of this experiment, MIP/Er^3+^/ZnS QDs can maintain their stability based on the protection of QD by SiO_2_ shells wrapped on the surface of QD. The effects of fluorescence concentration and target binding time on the fluorescence system are shown in Fig 7.

**Fig 8:**
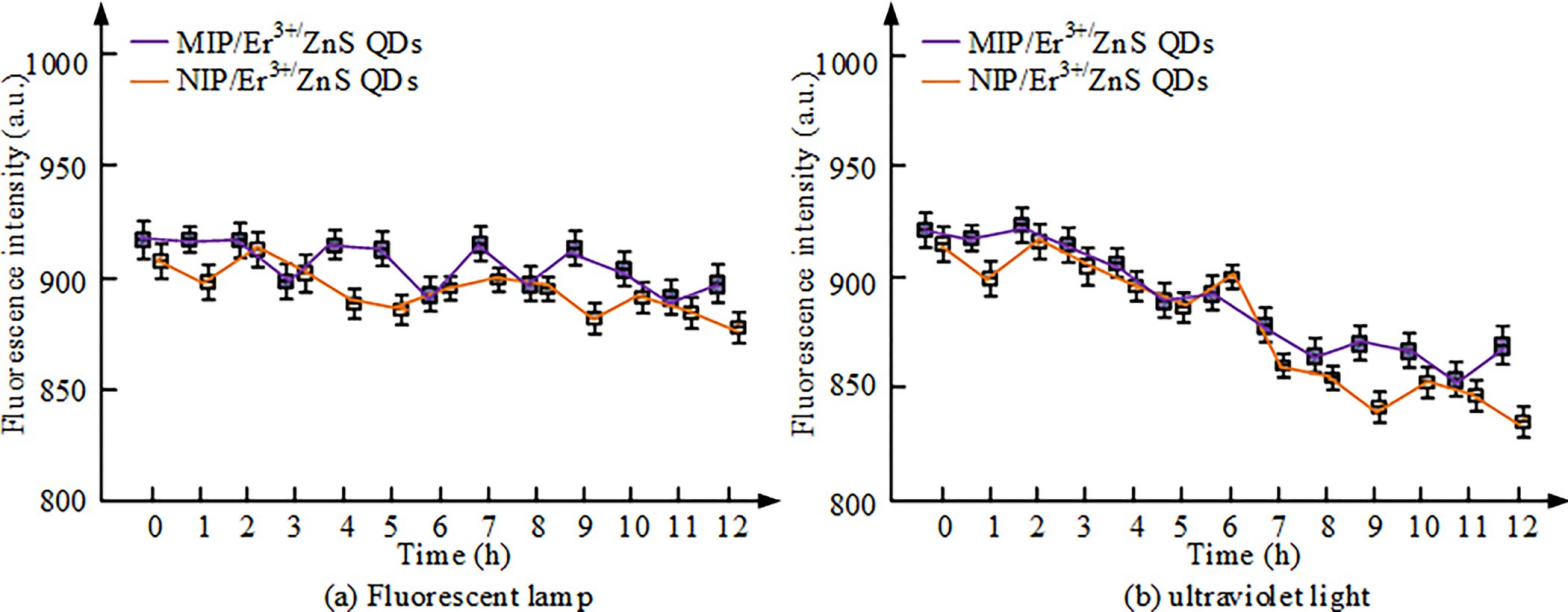
Changes in FI of MIP/Er^3+^/ZnS QDs and NIP/Er^3+^/ZnS QDs under light conditions. (a): Fluorescent lamp; (b): Ultraviolet light.

Fig 7(A) shows the effect of MIP/Er^3+^/ZnS QDs fluorescence concentration on the detection system. As the level of fluorescent substances is low, the FI is relatively weak, and as the level gradually increases, the FI will also increase. On the other hand, when the level of fluorescent substances is low, the fluorescence quenching rate is relatively small, and as the concentration increases, the fluorescence quenching rate also increases. In specific experiments, when the concentration of MIP/Er^3+^/ZnS QDs is 20 mg/L, the fluorescence quenching rate is the highest, while at a concentration of 30mg/L, relatively high FI can be obtained. To obtain a higher fluorescence quenching rate and relative FI, the optimal concentration of MIP/Er^3+^/ZnS QDs is determined to be 30mg/L. Fig 7(B) shows the changes in FI of MIP/Er^3+^/ZnS QDs after the fluorescence sensor is combined with the target substance. When CFX is added to MIP/Er^3+^/ZnS QDs, the FI of the solution rapidly decreases at the beginning, and then remains basically unchanged after about 10 minutes. This indicates that MIP/Er^3+^/ZnS QDs rapidly bind to CFX and cause fluorescence quenching, leading to a decrease in FI. This phenomenon can be explained by the presence of specific imprinted pores in the imprinted layer on the surface of MIP/Er^3+^/ZnS QDs that recognize CFX, while the imprinted layer is thin, allowing CFX to quickly bind and cause fluorescence quenching. Therefore, the molecularly imprinted fluorescence sensor MIP/Er^3+^/ZnS QDs can specifically bind to CFX in a short period, and the FI reaches stability after about 10 minutes. The FI changes of MIP/Er^3+^/ZnS QDs and NIP/Er^3+^/ZnS QDs under light conditions are shown in Fig 8.

**Fig 9:**
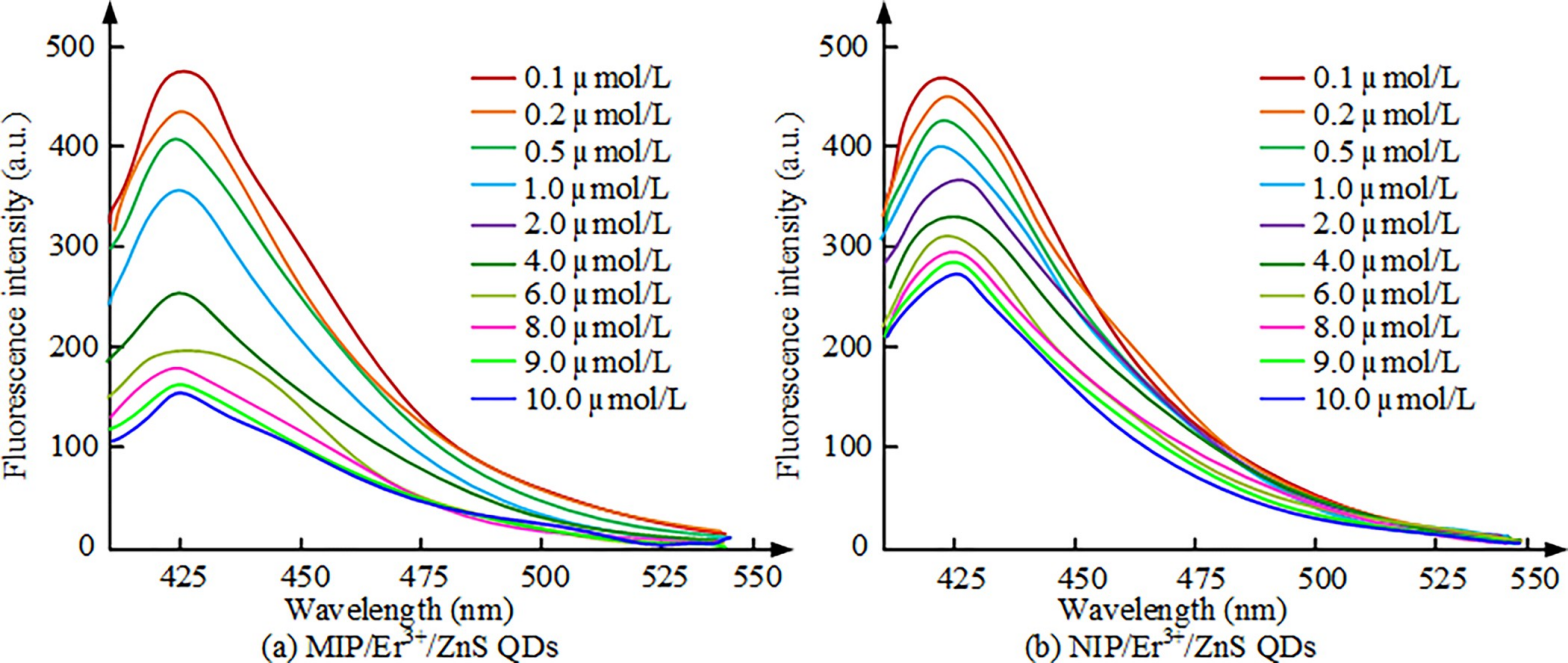
The effect of different concentrations of CFX on fluorescence detection luminescence spectra. (a): MIP/Er^3+^/ZnS QDs; (b): NIP/Er^3+^/ZnS QDs.

Fig 8 shows that the FI of MIP/Er^3+^/ZnS QDs and NIP/Er^3+^/ZnS QDs exhibit different trends under different lighting conditions. Under sunlight conditions, the FI of both remains basically constant, exhibiting a stable fluorescence signal. However, under continuous UV irradiation, the FI of both decreases, but the FI of MIP/Er^3+^/ZnS QDs decreases relatively less. This indicates that MIP/Er^3+^/ZnS QDs have better fluorescence stability and exhibit lower FI loss under UV irradiation. Therefore, MIP/Er^3+^/ZnS QDs have good stability and long-term storage performance, making them more reliable and practical in practical applications. The effect of various concentrations of CFX on the fluorescence detection luminescence spectrum is shown in Fig 9.

**Fig 10:**
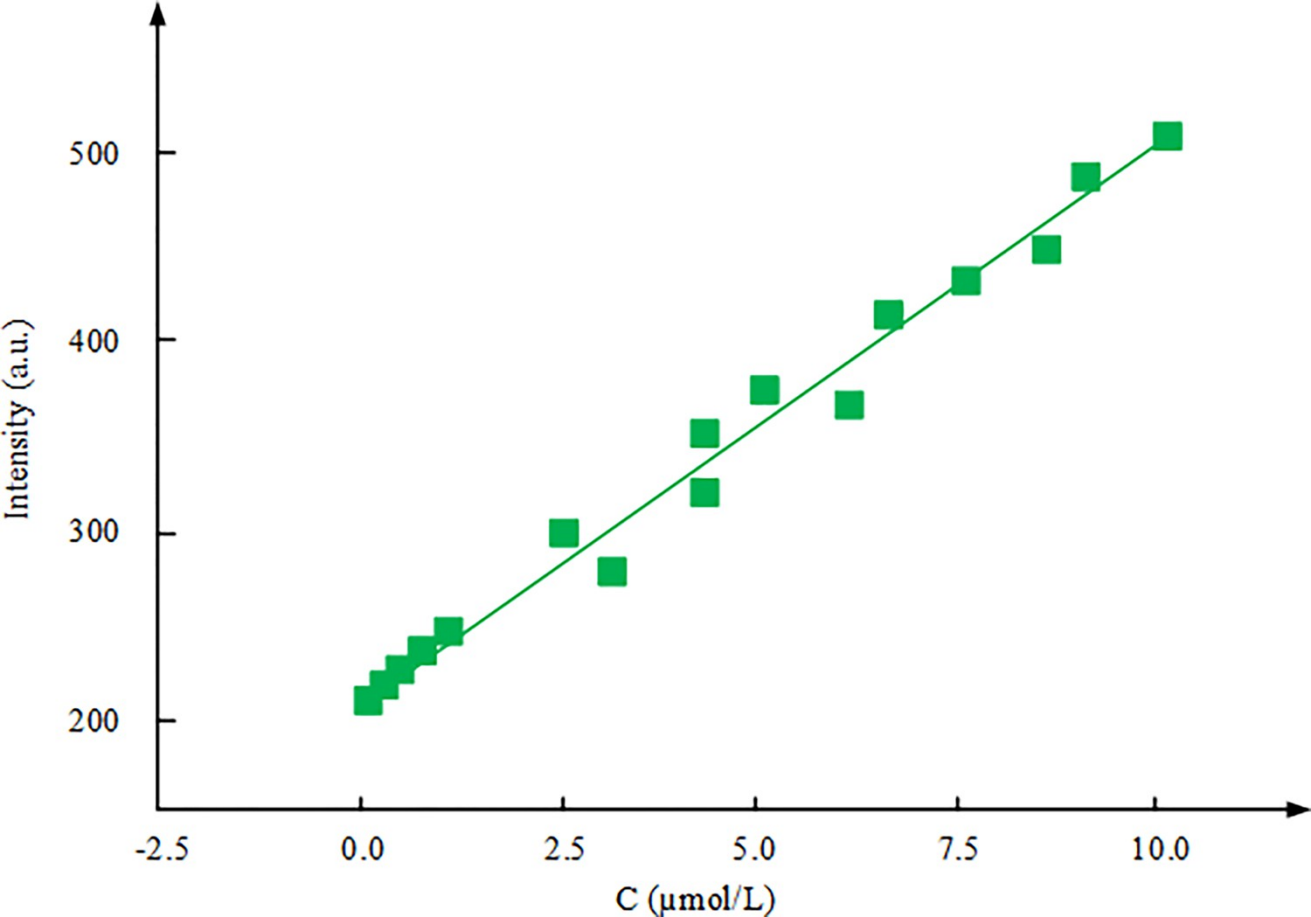
Linear regression equation between FI and CFX concentration.

In Fig 9, as the concentration of CFX grows, the FI of imprinted fluorescent sensors MIP/Er^3+^/ZnS QDs and non-imprinted fluorescent sensors NIP/Er^3+^/ZnS QDs gradually decreases. When the CFX concentration is within the range of 10μmol/L, the FI of both sensors undergoes significant changes. However, as the concentration of CFX increases to above 6μmol/L, the FI of MIP/Er^3+^/ZnS QDs slowly reduces. This indicates that MIP/Er^3+^/ZnS QDs have a higher fluorescence quenching effect, while the surface of NIP/Er^3+^/ZnS QDs does not have a specific binding site for CFX, resulting in a smaller change in FI. The linear regression equation between FI and CFX concentration is shown in Fig 10.

Fig 10 demonstrates the FI of the MIP/Er^3+^/ZnS QDs fluorescence sensor shows a linear relationship with the concentration of ciprofloxacin. When the concentration range is 0.1–10μmol/L, the relation in FI and concentration can be expressed by a linear equation. According to the limitations of the linear equation and a given 3-fold signal-to-noise ratio, the DL of the MIP/Er^3+^/ZnS QDs fluorescence sensor is experimentally calculated to be 31μmol/L.

### Selective detection analysis

**Fig 11:**
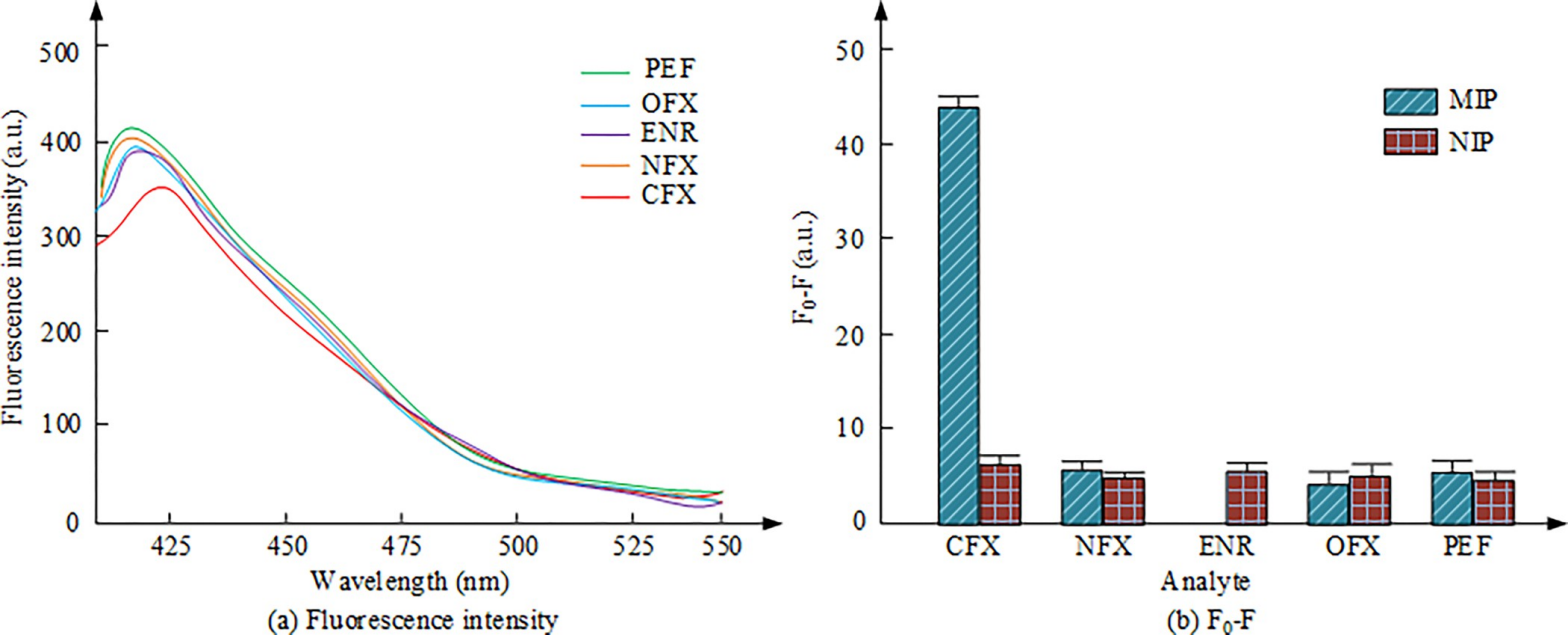
Effect of fluoroquinolone drugs on the determination of ciprofloxacin. (a): FI; (b): F_0_-F.

This experiment evaluated the selectivity of 30 mg/L MIP/Er^3+^/ZnS QDs by testing their response to 200x other fluoroquinolone drugs and 200x common coexisting ions. In the presence of 1μmol/L CFX, the effects of fluoroquinolone drugs such as norfloxacin (NFX), enrofloxacin (ENR), ofloxacin (OFX), and pefloxacin (OFX) were investigated in the experiment. By evaluating the detection response of these interfering substances, the selectivity of MIP/Er^3+^/ZnS QDs in actual sample determination could be determined, and its accuracy and reliability could be evaluated. The effect of fluoroquinolone on the determination of ciprofloxacin is shown in Fig 11.

Fig 11 shows that compared with other fluoroquinolones, ciprofloxacin has the most significant fluorescence sensitization effect on MIP/Er^3+^/ZnS QDs, indicating that the sensor has specificity for ciprofloxacin. This specificity can be attributed to the specific recognition of the MIP cavity with ciprofloxacin [25]. It is worth noting that within the allowable range of 5% error, common coexisting ions have no effect on the determination of MIP/Er^3+^/ZnS QDs sensors. In summary, ciprofloxacin has specificity and good detection impact in MIP/Er^3+^/ZnS QDs sensors, making it an effective fluorescent sensitizer for the determination of target molecules.

### Sample testing and analysis

For estimating the capability of MIP/Er^3+^/ZnS QDs in the actual determination of ciprofloxacin, experiments were conducted on milk samples. Firstly, the blank milk sample was tested, but the presence of ciprofloxacin was not detected. To conduct spiked recovery experiments, various concentrations of ciprofloxacin were added to the samples and three measurements were performed on each sample. The detection results of ciprofloxacin in milk samples are shown in Table 3.

**Table 3:** Detection results of ciprofloxacin in milk samples.

Number	Added (μmol/L)	Found (μmol/L)	Recovery (%)	Relative standard deviation (%)
1	0.10	0.100	99.8	1.9
2	0.20	0.200	99.9	1.7
3	0.50	0.499	99.7	2.1
4	0.80	0.809	101.1	2.8
5	1.00	1.023	102.3	3.5
6	2.00	2.064	103.2	3.2
7	3.00	2.991	99.7	2.1
8	4.00	4.012	100.3	2.2
9	5.00	5.175	103.5	2.9

In Table 3, the recovery rate of ciprofloxacin in milk samples determined by MIP/Er^3+^/ZnS QDs is 99.7% to 103.5%, with a relative standard deviation of less than 5%. This indicates that MIP/Er^3+^/ZnS QDs can accurately determine the content of ciprofloxacin in milk samples, and have good repeatability and stability. The spiked recovery experiment confirms that the determination results of MIP/Er^3+^/ZnS QDs in milk samples are close to theoretical values. The variability between the measured values is small, further proving the selectivity and accuracy of the sensor for ciprofloxacin. This study analyzes distilled water, tap water, and aquaculture water samples using MIP/Er^3+^/ZnS QDs to evaluate their applicability in actual water samples. The experimental results can help understand the pollutant content in environmental water samples, providing reference for protecting the environment and human health. For ensuring the precision experimental results, multiple repeated experiments are conducted and the average detection concentration is calculated to reduce experimental errors. The results of environmental water sample testing are shown in Table 4.

**Table 4:** Environmental water sample testing results.

Sample type	Number	Added (μmol/L)	Found (μmol/L)	Recovery (%)
Distilled water	1	0.00	-	-
	2	0.05	0.0498	99.6
	3	0.10	0.1023	103.4
	4	0.25	0.2485	99.4
	5	0.50	0.4495	89.9
Tap-water	1	0.00	-	-
	2	0.05	0.0511	102.2
	3	0.10	0.1034	103.4
	4	0.25	0.2573	102.9
	5	0.50	0.4367	87.3
Aquaculture water	1	0.00	-	-
	2	0.05	0.0517	103.3
	3	0.10	0.1058	105.8
	4	0.25	0.2565	102.6
	5	0.50	0.4155	83.1

Table 4 shows that MIP/Er^3+^/ZnS QDs exhibit excellent recovery rates for CFX detection in different types of water samples (distilled water, tap water, and aquaculture water). Within the linear concentration range (0–0.50μmol/L), this molecularly imprinted fluorescence sensor can effectively and accurately detect CFX in actual environmental water. The results indicate that MIP/Er^3+^/ZnS QDs are a reliable tool for rapid detection and monitoring of CFX in environmental water samples. However, there is a slight difference in the recovery rate between tap water and aquaculture water, and further research and optimization are needed in the future. To verify the superiority of the fluorescent sensor MIP/Er^3+^/ZnS QDs, existing fluorescent sensors are compared in the experiment. The comparison of detection results of different fluorescent sensors is shown in Table 5. The results show that among these sensors, the MIP/Er^3+^/ZnS QDs fluorescence sensor exhibits the best performance, with a lower DL (31 nmol/L) and a wider linear range (0–0.50 μmol/L). This makes MIP/Er^3+^/ZnS QDs fluorescence sensors highly sensitive and accurate in practical applications.

**Table 5:** Comparison of the detection results for the different fluorescent sensors.

Sensor	Detection limit	Linear range
MIP/Er^3+^/ZnS QDs	31 nmol/L	0–0.50 μmol/L
Eu FITC ratio fluorescence sensor	50 nmol/L	0–0.25 μmol/L
COF-AIECL sensor	60 nmol/L	0–0.20 μmol/L
Eu^3+^ reference carbon dot ratio fluorescence sensor	55 nmol/L	0–0.25 μmol/L
Carbon nitride/titanium carbide photoelectric detection sensor	48 nmol/L	0–0.30 μmol/L

Based on the above research results, this study prepared MIP/Er^3+^/ZnS QD materials using co-precipitation method, and characterized their morphology, spectra, and fluorescence properties. The results indicated that the material had a spherical shape and uniform distribution, with good dispersibility and stability. In addition, the sensor prepared in this experiment could achieve maximum FI under optimal conditions (pH = 5, Er^3+^ concentration of 1.2 mmol/L, incubation time of 20 minutes), and remained stable within a detection time of 40 minutes. The sensor had high specificity and sensitivity for ciprofloxacin, with a DL of 31 nmol/L and a linear range of 0–0.5 μmol/L. The actual sample test results showed that the sensor had good recovery and stability in detecting ciprofloxacin in milk and environmental water samples, exhibiting excellent selectivity and accuracy. Compared with other fluorescent sensors, this sensor had higher sensitivity and wider linear range, demonstrating its advantages in practical applications. In summary, the molecularly imprinted fluorescence sensor was a reliable and practical tool that can be widely used for rapid detection and monitoring of fluoroquinolone drugs such as ciprofloxacin.

## Conclusion

This study aimed to prepare a fluorescent sensor based on molecular imprinting technology to achieve selective detection of ciprofloxacin. To improve the FI of QD and enhance the sensitivity of sensors for ciprofloxacin detection, the rare earth metal Er^3+^ was introduced and added to ZnS QD. During the experimental process, the MIP/Er^3+^/ZnS QDs sensor was first prepared. Then, the study optimized the experimental conditions of the sensor and determined the optimal linear range for the determination of ciprofloxacin from 0.1 to 10 μmol/L. Through the analysis of experimental results, the study found that the sensor has excellent selectivity for ciprofloxacin. In milk samples, the recovery rates of MIP/Er^3+^/ZnS QDs ranged from 99.7% to 103.5%, and the relative standard deviation was less than 5%. This study successfully combined molecular imprinting technology with fluorescence sensors, enabling the sensor to have higher selectivity and sensitivity in the detection of ciprofloxacin. This provides an effective detection method for environmental monitoring and food safety. However, the recovery rate of the sensor in tap water samples is slightly lower than that in distilled water and aquaculture water samples. This indicates that the detection effect of the sensor in tap water samples is slightly poor and needs further research and optimization. In addition, the sensor has a small change in FI when detecting low concentrations of ciprofloxacin, and the detection sensitivity needs to be improved. Future research can consider optimizing the design and preparation methods of sensors to further improve their detection sensitivity and selectivity. Meanwhile, efforts can be made to apply the sensor to a wider range of environmental water samples to evaluate its applicability in practical environmental monitoring.

## Supporting information

S1 File (DOC)
