# Supplementary material for: Preparation of molecular imprinted fluorescence sensor based on Er3+/ZnS QDs and its selective detection of ciprofloxacin in various matrices
Source: PLoS One. 2024 Dec 19;19(12):e0312156. doi: 10.1371/journal.pone.0312156 (PMC11658606; doi:10.1371/journal.pone.0312156)
Supplement: S1 File — (DOC) [file pone.0312156.s001.doc]

**The data in Figure 3**

| Wavenumber (cm^-1^) | Transmittance (%) | | |
| --- | --- | --- | --- |
|  | ZnS QDs | Er^3+^/ZnS QDs | MIP/Er^3+^/ZnS QDs |
| 500 | 84.1 | 73.2 | 66.1 |
| 1000 | 79.9 | 73.6 | 62.7 |
| 1500 | 78.2 | 69.3 | 61.7 |
| 2000 | 79.6 | 71.2 | 62.9 |
| 2500 | 78.4 | 70.6 | 61.9 |
| 3000 | 78.5 | 69.9 | 60.6 |
| 3500 | 84.2 | 70.1 | 65.9 |
| 4000 | 84.5 | 74.7 | 65.1 |

**The data in Figure 4**

| 1. Absorbance (a.u.) | | | 1. Intensity (a.u.) | | | | | |
| --- | --- | --- | --- | --- | --- | --- | --- | --- |
| Wavelength (nm) | ZnS QDs | Er^3+^/ZnS QDs | Wavelength (nm) | ZnS QDs | Er^3+^/ZnS QDs | NIP/Er^3+^/ZnS QDs | MIP/Er^3+^/ZnS QDs 1 | MIP/Er^3+^/ZnS QDs 2 |
| 260 | 1.22 | 0.98 | 325 | 2.1 | 11.9 | 10.3 | 17.3 | 13.8 |
| 280 | 0.92 | 0.69 | 375 | 19.9 | 18.8 | 21.2 | 83.2 | 39.2 |
| 300 | 0.42 | 0.38 | 425 | 169.8 | 281.1 | 358.6 | 681.3 | 317.6 |
| 320 | 0.01 | 0.02 | 475 | 51.3 | 89.5 | 119.9 | 240.1 | 99.9 |
| 340 | 0.00 | -0.02 | 525 | 12.4 | 11.1 | 29.3 | 42.2 | 20.7 |

**The data in Figure 5**

| 1. Er^3+^ concentration | | 1. pH | |
| --- | --- | --- | --- |
| C_Er_ (mmol L^-1^) | Intensity (a.u.) | pH | F-F_0_ (a.u.) |
| 0 | 389.2±13.2 | 4 | 110.6±4.3 |
| 1 | 735.2±26.3 | 5 | 149.9±3.9 |
| 2 | 771.3±9.8 | 6 | 134.8±3.2 |
| 3 | 742.1±11.8 | 7 | 110.1±3.5 |
| 4 | 721.6±20.9 | 8 | 79.9±3.6 |
| 5 | 719.3±29.8 | 9 | 34.7±3.7 |
| 6 | 691.3±11.5 | 10 | 10.2±2.4 |
| 7 | 675.4±12.1 | / | / |
| 8 | 628.7±27.9 | / | / |
| 9 | 602.3±13.7 | / | / |
| 10 | 573.6±12.8 | / | / |

**The data in Figure 6**

| (a) Incubation time | | (b) Fluorescence stability | |
| --- | --- | --- | --- |
| Time (min) | Intensity (a.u.) | Time (min) | Intensity (a.u.) |
| 0 | 350.0±3.3 | 0 | 204.3±5.7 |
| 20 | 380.4±3.4 | 5 | 192.4±5.9 |
| 40 | 385.1±3.5 | 10 | 205.1±6.8 |
| 60 | 386.2±3.1 | 15 | 200.1±6.2 |
| 80 | 387.1±3.4 | 20 | 207.6±6.1 |
| 100 | 387.9±3.1 | 25 | 211.3±5.6 |
| 120 | 386.8±3.6 | 30 | 203.7±4.2 |
| / |  | 35 | 200.6±5.2 |
| / |  | 40 | 209.4±4.1 |

**The data in Figure 7**

| (a) Incubation time  F-F_0_ (a.u.) | | | (b) Fluorescence stability  Fluorescence intensity (a.u.) | | | | |
| --- | --- | --- | --- | --- | --- | --- | --- |
| Time (min) | F-F_0_ | Relative intensity | Time (min) | 0 min | 5 min | 10 min | 30 min |
| 5 | 0.51±0.09 | 0.32±0.10 | 520 | 951 | 493 | 331 | 284 |
| 10 | 0.63±0.07 | 0.58±0.11 | 540 | 782 | 398 | 271 | 246 |
| 15 | 0.78±0.09 | 0.48±0.12 | 560 | 457 | 221 | 173 | 132 |
| 20 | 0.81±0.11 | 0.54±0.10 | 580 | 226 | 119 | 71 | 58 |
| 25 | 0.76±0.07 | 0.67±0.13 | 600 | 116 | 82 | 54 | 25 |
| 30 | 0.69±0.06 | 0.73±0.14 | 620 | 73 | 47 | 26 | 15 |
| 35 | 0.62±0.05 | 0.79±0.09 | 640 | 57 | 39 | 24 | 13 |
| 40 | 0.48±0.04 | 0.87±0.15 | / | / | / | / | / |
| 45 | 0.41±0.12 | 0.91±0.11 | / | / | / | / | / |
| 50 | 0.36±0.09 | 1.00±0.01 | / | / | / | / | / |

**The data in Figure 8**

| Tmie (h) | Fluorescence intensity (a.u.) | | | |
| --- | --- | --- | --- | --- |
|  | Fluorescent lamp | | Ultraviolet light | |
|  | MIP/Er^3+/^ZnS QDs | NIP/Er^3+/^ZnS QDs | MIP/Er^3+/^ZnS QDs | NIP/Er^3+/^ZnS QDs |
| 1 | 921±21 | 918±20 | 915±23 | 913±22 |
| 2 | 920±22 | 889±22 | 914±21 | 904±20 |
| 3 | 922±20 | 921±21 | 914±25 | 913±19 |
| 4 | 901±19 | 905±20 | 905±21 | 901±21 |
| 5 | 910±20 | 891±18 | 898±25 | 899±20 |
| 6 | 889±18 | 900±17 | 900±24 | 904±18 |
| 7 | 909±19 | 901±19 | 879±23 | 871±19 |
| 8 | 900±20 | 901±21 | 859±20 | 855±21 |
| 9 | 914±20 | 879±22 | 875±24 | 856±20 |
| 10 | 900±19 | 889±21 | 873±21 | 852±22 |
| 11 | 882±27 | 883±19 | 852±20 | 850±20 |
| 12 | 901±20 | 877±17 | 873±25 | 834±21 |

**The data in Figure 9**

| Wavelength (nm) | Fluorescence intensity (a.u.) | | | | | |
| --- | --- | --- | --- | --- | --- | --- |
|  | MIP/Er^3+/^ZnS QDs | | | NIP/Er^3+/^ZnS QDs | | |
|  | 0.1 µmol/L | 4.0 µmol/L | 1.0 µmol/L | 0.1 µmol/L | 4.0 µmol/L | 1.0 µmol/L |
| 425 | 476 | 258 | 151 | 468 | 328 | 274 |
| 450 | 209 | 152 | 98 | 275 | 213 | 151 |
| 475 | 128 | 77 | 49 | 117 | 109 | 68 |
| 500 | 54 | 28 | 24 | 51 | 47 | 26 |
| 525 | 25 | 13 | 4 | 23 | 21 | 19 |

**The data in Figure 10**

| C (µmol/L) | Intensity (a.u.) |
| --- | --- |
| 0.0 | 210.2 |
| 2.5 | 299.4 |
| 5.0 | 375.1 |
| 7.5 | 417.6 |
| 10.0 | 503.7 |

**The data in Figure 11**

| Fluorescence intensity (a.u.) | | | | | | F-F_0_ (a.u.) | | |
| --- | --- | --- | --- | --- | --- | --- | --- | --- |
| Wavelength (nm) | PEF | OFX | ENR | NFX | CFX | Analyte | MIP | NIP |
| 425 | 492 | 463 | 478 | 479 | 422 | CFX | 44 | 7 |
| 450 | 256 | 237 | 239 | 242 | 219 | NFX | 6 | 5 |
| 475 | 134 | 118 | 122 | 121 | 122 | ENR | 0 | 6 |
| 500 | 51 | 47 | 51 | 47 | 52 | OFX | 4 | 5 |
| 525 | 26 | 33 | 25 | 52 | 51 | PEF | 6 | 4 |
